# Homozygous 896delT (c.764del) in Somali–American Siblings With Cystic Fibrosis

**DOI:** 10.1155/crpu/1148927

**Published:** 2026-04-24

**Authors:** Disi Chen, Elinor Langfelder-Schwind, Denise Kay, Zafer Soultan

**Affiliations:** ^1^ Division of Pediatric Pulmonary Medicine, SUNY Upstate Medical University, Syracuse, New York, USA, upstate.edu; ^2^ Department of Pulmonary Medicine, Lenox Hill Hospital at Northwell Health, New York City, New York, USA; ^3^ Division of Genetics, New York State Department of Health, Albany, New York, USA, health.ny.gov

## Abstract

Cystic fibrosis is an autosomal recessive condition historically reported to be predominantly in those of European ancestry; however, more cases of CF are reported in those with non‐European ancestry as we improve diagnostic awareness and aptitude. We report two cases of Somali siblings with rare *CFTR* 896delT (c.764del; p.Ile255ThrfsX6; dbSNP rs2485020167; ClinVar ID 2682475; NM_000492.4:c.764del) homozygous variants who manifested classic CF lung and hepatic disease. They presented with elevated immunoreactive trypsinogen on newborn screen but required exon sequencing to confirm their rare variant. This type of variant causes a frameshift that leads to an early stop codon; therefore, there is currently no available disease‐modulating therapy. Our cases highlight the clinical phenotype of this variant and increase awareness of the importance of inclusivity in our current CF screening methods.

## 1. Introduction

The incidence of cystic fibrosis (CF) varies by geography and ancestral group [1]; it is estimated at 1 in 4400 newborns in the United States and 1 in 17,000 African Americans [2]. Since the identification of the Cystic Fibrosis Transmembrane Conductance Regulator (*CFTR*) gene in 1989, over 2000 variants have been reported; 1185 have been characterized as CF‐causing (http://www.cftr2.org). As new variants continue to be discovered, there is increasing recognition of racial‐ethnic disparities in diagnosis and treatment of CF [3, 4].

To ensure equitable detection of babies with CF in New York State′s (NYS) diverse population, the NYS newborn screening (NBS) program has undergone several algorithm and DNA panel changes, including transition from a two‐tier immunoreactive trypsinogen/DNA (IRT‐DNA) algorithm evaluating 39 *CFTR* variants, to a three‐tier IRT‐DNA‐SEQ algorithm in December 2017 [5]. As of November 2024, infants with the Top 5% IRT of the day undergo second‐tier panel analysis, which targets 1018 variants as of August 2025 [6]. Infants with two *CFTR* variants are referred for sweat testing and specialist consultation. Infants with one *CFTR* panel variant or no variants but with very high IRT (VHIRT; Top 0.1%) undergo third‐tier *CFTR* gene sequence analysis. Sequencing allows the detection of rare pathogenic variants not targeted by commercial panels. With the previous IRT‐DNA algorithm, infants with one to two panel variants or VHIRT were considered screen positive and referred. Further details of the algorithm have been described by Wilschanski [7].

We present clinical and genetic findings of homozygosity for a rare CF‐causing variant in a pair of Somali–American siblings with discordant CF NBS results.

## 2. Case Report

### 2.1. Sibling A

Sibling A is now a 13‐year‐old female. She was born full‐term without meconium ileus (MI) to native Somalian parents who are first cousins. She screened positive for CF in 2010 and was referred by NYS NBS based on VHIRT (IRT = 138 ng/mL) with no panel variants. Her CF diagnosis was confirmed at 1 month of age via sweat chloride testing (90/97 mmol/L). She was subsequently found to be homozygous for the variant 896delT (c.764del; p.Ile255ThrfsX6; dbSNP rs2485020167; ClinVar ID 2682475; NM_000492.4:c.764del) on *CFTR* clinical exon sequencing assay (Genzyme Genetics, Westborough, Massachusetts, United States).

She was determined to be pancreatic insufficient (fecal elastase < 15 mcg/g) and had difficulty maintaining BMI between 25th and 50th centile for most of her life. Her sputum cultures have grown *Actinobacterium*, *Pseudomonas aeruginosa*, *MSSA*, and *Mycobacterium avium* thus far. All were successfully eradicated aside from the *M. avium*, for which our team determined clinical monitoring was appropriate.

Her spirometry (percent predicted forced expiratory volume in 1 s [ppFEV_1_]) gradually declined over the years to mid‐60′s by the age of 13 years. She has evidence of hepatic disease with ALT 82 U/L, AST 73 U/L, and gamma‐glutamyl transferase (GGT) 46 U/L. Her recent CT showed mild bronchiectasis bilaterally, two lung nodules of unclear etiology, and hepatomegaly with diffuse hepatic steatosis.

### 2.2. Sibling B

Sibling B, now a 9‐year‐old male with the same parents as Sibling A, was born at full‐term without complication and did not have MI or delayed meconium passage. NBS was negative; IRT was above the 5%ile (48 ng/mL), but none of the 39 panel variants was found, and the IRT was not high enough to be considered screen positive based on VHIRT (0.1%ile). The family had not been seen in clinic until Sibling B was admitted at 3 months of age with diarrhea and failure to thrive. Sweat chloride testing was positive (90/95 mmol/L), and he was pancreatic insufficient (fecal elastase < 15 mcg/g). His *CFTR* clinical exon sequencing assay confirmed homozygosity for 896delT.

Sibling B was hospitalized three times for pulmonary exacerbations by the age of 9 years. At 2 years of age, he was diagnosed with CF‐related liver disease, with recent liver function testing revealing ALT 169 U/L, AST 91 U/L, GGT 62 U/L. Other comorbidities include chronic otitis media requiring ear tubes, eczema, and multiple food allergies. Sputum cultures have grown *Actinobacterium*, *P. aeruginosa*, MSSA, *Elizabethkingia meningoseptica*, and *Chryseobacterium indologenes*.

His ppFEV_1_ was initially in the high‐100′s and is now in the mid‐80′s. Recent chest CT showed peribronchiolar thickening and marginal bronchiectasis extending to the lower lobes. An abdominal sonogram at the same age showed diffusely heterogeneous enlarged liver. The parents of these siblings received genetic counseling through the CF clinic provider at multiple points of care.

## 3. Discussion

Here, we present siblings with classic CF disease caused by homozygous 896delT (c.764del; p.Ile255ThrfsX6). This variant, which was not described in any worldwide CF databases when initially reported (now registered in ClinVar), is a single nucleotide deletion that leads to a frameshift, resulting in a premature stop codon that leads to an absence of CFTR protein as seen in Class I type variants. Class I variants result in classic CF clinical presentations including severe respiratory disease and pancreatic insufficiency as described in our siblings [7].

The NYS NBS program implemented a customizable second‐tier *CFTR* panel in 2019 that included pathogenic variants (including 896delT) found in the homozygous state in screen‐positive NYS patients with CF but had not been reported in the CFTR2 database [8]. The 896delT variant was entered into the CFTR2 database and characterized as CF‐causing in 2023.

Although F508del remains the most common *CFTR* variant in the United States and most of Europe, other variants are more predominant in different parts of Africa [9]. People of African American ancestry are more likely to have later diagnosis compared with their non‐Hispanic White counterparts partially due to having rarer *CFTR* variants that as a result are either not included on an initial screening panel or are novel [4, 10]. Studies have shown inherent disparities when comparing time from detection on NBS for CF to confirmatory testing and/or specialist encounter, with better outcomes in non‐Hispanic White individuals as compared with non‐White individuals [3].

Delays in diagnosis lead to delayed initiation of treatment including effective modulators and can lead to irreparable lung damage secondary to airway inflammation and bacterial infection that can occur as early as the first 4 weeks of life [11, 12]. The currently available modulators have been shown to significantly improve lung function, nutritional status, and quality of life [13, 14]. The starting age for initiating modulators is gradually decreasing as studies have shown improved clinical outcomes with good safety measures [15]. Despite these effective therapies, there is still a significant proportion of patients with CF, including Siblings A and B, whose variants do not qualify for available modulators and remain on traditional symptom‐based therapies.

This case report highlights the need to evaluate symptomatic individuals and siblings of people with CF, particularly those with rare *CFTR* variants that are not included on CF NBS panels, and not to rely on normal NBS results, especially if there is increased risk for CF due to family history. NBS is not diagnostic; NBS is a population screen known to miss some infants with CF [16]. Sibling A was referred for CF evaluation and diagnosed at 1 month of age due to New York′s VHIRT referral category, which is not utilized by most NBS programs. VHIRT has a low positive predictive value, but Kay et al. found that babies identified via VHIRT were more likely to be from minoritized racial and ethnic backgrounds [3]. Sibling B′s diagnosis was delayed due to several factors, including a false negative NBS due to a limited second tier genetic panel at the time of testing and loss of communication between the CF clinic, the parents, and the pediatrician; this lead to the failure to develop a birth plan that included CF evaluation within 1 month of age regardless of the NBS result. NYS provides access to DNA analysis for siblings with a normal NBS result by special request with informed parental consent.

Addressing disparities in positive NBS rates between those of different racial and ethnic backgrounds and delays in CF diagnosis requires ongoing efforts, in part by expanding our knowledge of *CFTR* variants.

## Funding

No funding was received for this manuscript.

## Consent

Verbal informed consent for publication of their clinical details was obtained from the parents of the patients. Waiver of written consent was obtained.

## Conflicts of Interest

The authors declare no conflicts of interest.

## Data Availability

The data that support the findings of this study are available from the corresponding author upon reasonable request.
